# Novel estimation of African swine fever transmission parameters within smallholder villages in Lao P.D.R

**DOI:** 10.1007/s11250-024-04012-z

**Published:** 2024-05-17

**Authors:** Nina Matsumoto, Michael P. Ward, Tariq Halasa, Kathrin Schemann, Syseng Khounsy, Bounlom Douangngeun, Watthana Thepagna, Phouvong Phommachanh, Jarunee Siengsanan-Lamont, James R. Young, Jenny-Ann L. M. L. Toribio, Russell D. Bush, Stuart D. Blacksell

**Affiliations:** 1 Sydney School of Veterinary Science, Faculty of Science, The University of Sydney, Camden, NSW 2570 Australia; 2 Section of Animal Welfare and Disease Control, Department of Veterinary and Animal Sciences, Faculty of Health and Medical Sciences, University of Copenhagen, Copenhagen, Denmark; 3 Sydney Informatics Hub, The University of Sydney, Camperdown, NSW Australia; 4 National Animal Health Laboratory, Department of Livestock and Fisheries, Ministry of Agriculture and Forestry, Vientiane, Lao People’s Democratic Republic; 5 Mahidol-Oxford Tropical Medicine Research Unit, Faculty of Tropical Medicine, Mahidol University, Bangkok, Thailand; 6 Centre for Tropical Medicine & Global Health, Nuffield Department of Medicine, University of Oxford, Oxford, UK; 7 Lao-Oxford-Mahosot Hospital-Wellcome Trust Research Unit (LOMWRU), Mahosot Hospital, Vientiane, Lao People’s Democratic Republic

**Keywords:** African swine fever, Smallholder village, Lao, Disease modelling, Pig, Transmission parameters, Approximate Bayesian computation

## Abstract

African Swine Fever (ASF) disease transmission parameters are crucial for making response and control decisions when faced with an outbreak, yet they are poorly quantified for smallholder and village contexts within Southeast Asia. Whilst disease-specific factors − such as latent and infectious periods − should remain reasonably consistent, host, environmental and management factors are likely to affect the rate of disease spread. These differences are investigated using Approximate Bayesian Computation with Sequential Monte-Carlo methods to provide disease parameter estimates in four naïve pig populations in villages of Lao People’s Democratic Republic. The villages represent smallholder pig farmers of the Northern province of Oudomxay and the Southern province of Savannakhet, and the model utilised field mortality data to validate the transmission parameter estimates over the course of multiple model generations. The basic reproductive number between-pigs was estimated to range from 3.08 to 7.80, whilst the latent and infectious periods were consistent with those published in the literature for similar genotypes in the region (4.72 to 6.19 days and 2.63 to 5.50 days, respectively). These findings demonstrate that smallholder village pigs interact similarly to commercial pigs, however the spread of disease may occur slightly slower than in commercial study groups. Furthermore, the findings demonstrated that despite diversity across the study groups, the disease behaved in a consistent manner. This data can be used in disease control programs or for future modelling of ASF in smallholder contexts.

## Introduction

African Swine Fever (ASF) is a viral, haemorrhagic disease of domestic pigs and wild S*uidae* species of high morbidity and mortality, causing up to 100% case fatalities in naïve populations. In June 2019 ASF appeared in villages of southern Lao People’s Democratic Republic (Lao PDR, or henceforth Laos or Lao), where it devastated the smallholder pig populations (FAO 2020; Matsumoto et al. 2021). Over the next six months, the disease spread to every province in Laos (FAO 2020). All sectors of the pig industry were affected, with spill over events recorded in wild boar populations in the north-eastern Province of Houaphan (Denstedt et al. 2021). Previous outbreak investigations provided estimates of the ASF outbreak parameters such as the average period from the development of clinical signs to death (4.40 days, SD 6.1 days); and the interquartile ranges – or period over which 50% of all deaths occurred – ranging from 5.50 to 35 days (Matsumoto et al. 2021, 2023).

Disease transmission parameters are valuable in outbreak scenarios for decision making, in-depth modelling and analysis of socioeconomic impacts on the human and animal populations (Keeling and Rohani 2011). An example of a disease transmission parameter is the basic reproductive number or R_0_. The R_0_ is the average number of new infections caused by a single infected animal in a susceptible population (Dohoo et al. 2009). Estimates for R_0_ can be made between-pigs, between-pens (within a farm), between-farms or even between-villages, depending on the epidemiologic unit (Dohoo et al. 2009). All estimates for the between-pig R_0_ of the Georgia 2007 ASF strain concern commercial breed, intensively managed pigs, and range from 2.80 to 16.20 in experimental and field studies (Guinat et al. 2016a, 2018; Li et al. 2021). In the case of Laos, the 2019 ASF outbreak was the first recorded in the nation, and the local pig population were fully susceptible, but there is no published information about the R_0_ or transmission rate (*β*, sometimes called effective contact rate) for ASF virus (ASFV) between Lao pigs. The nearby Vietnamese outbreak during 2019 featured an isolate identical to Georgia 2007 and China 2018 isolates and therefore assumptions about transmission parameters based on the Lao 2019 outbreak data should be related to these strains (Le et al. 2019).

Different management techniques, pig health and biosecurity are all factors that create variations in the rate at which the same disease spreads from one pig to the next, as observed with differences in ASF outbreaks involving Georgia 2007 strain in Russia (Guinat et al. 2018). The pig rearing systems of Laos display marked diversity of management and biosecurity factors from the smallholder to the commercial scale (Phengsavanh et al. 2011; Holt et al. 2019). Smallholders typically own one to fifteen pigs raised in free-ranging contexts with variable production levels and health inputs. These pigs are traditional Lao breeds, with sporadic mixing events occurring with wild boar (Matsumoto et al. 2021, 2023). Knowledge, attitudes, and practices relating to livestock vary considerably in Laos due to the ethnic heterogeneity of the individuals rearing them (Nampanya et al. 2017; Holt et al. 2019; Matsumoto et al. 2021, 2023). Ethnic groups such as the Lao-Tai may prioritise penned fattening of individual pigs; others like the Hmong-Mien allow free-ranging, scavenging systems (Phengsavanh et al. 2010, 2011). These variations in management lead to difficulties in making population-level assumptions about smallholder Lao pigs for modelling purposes.

Veterinary resources of developing nations are often stretched in an outbreak, making the collection of detailed clinical data for use in future modelling a challenge when simultaneously carrying out disease prevention and control activities, preliminary disease investigations and stakeholder educational activities (Matsumoto et al. 2021). Parameter estimation techniques like Approximate Bayesian Computation with Sequential Monte Carlo (ABC-SMC) can be utilised to make the best use of available mortality data to understand the transmission parameter values that explain the spread of an ASF outbreak. There is currently no published ASF between-pigs R_0_ estimates in the Southeast Asian smallholder village context. The objective of this work is to provide the first published estimates of crucial transmission parameters for ASF using real-world Lao field data and previously published information about the Georgia 2007 and China 2018 strains of ASFV.

## Materials and methods

The parameter estimation process involved building an ABC-SMC model of four separate study villages, to determine the transmission parameters between-pigs in each separate pig population. In this model, the outputs of a between-pig disease spread model were compared to field mortality data and gradually refined over multiple generations until the estimates stabilised and provided a good, simulated fit to the real data.

### Field mortality data collection

#### Village selection

The study included two villages each (n = four) from Savannakhet province and Oudomxay province. These were chosen purposively, as they had confirmed ASF cases in 2019 and local animal health authorities suggested they were representative of regional outbreak locations based in the Northern and Southern regions of Laos (Matsumoto et al. 2021, 2023).

#### Collection methods

Mortality and clinical infectious period data were collected using surveys described by Matsumoto et al. (Matsumoto et al. 2021, 2023). The Lao Department of Livestock and Fisheries (DLF) consulted with the local Provincial and District Agriculture and Forestry Office (PAFO and DAFO, respectively) staff to select villages and contact the Village Chiefs prior to the survey dates. Data describing each of the villages can be found in Tables 1 and 2.

**Table 1:** Details on survey responses in villages selected for modelling

Variable^1^	Densateung, Savannakhet	Phouphanang-Khampia, Savannakhet	Doneant, Oudomxay	Huaylerm, Oudomxay
No. survey responses	24	25	14	14
Free-ranging or partial free-ranging pig housing (%)	59.2%^2^	59.2%^2^	14.9%^2^	14.9%^2^
Containment system (own or communal pens) housing (%)	8.0%^2^	8.0%^2^	85.0%^2^	85.0%^2^
Pig losses per household ^2^	6 piglets (7.25)^3^ 1 sow (2)^3^	4 piglets (6)^3^ 1 sow (1)^3^	3 (2.4)	4.3 (2.6)
Outbreak period (days)	65	33	90	103
Median epidemic day	1 June 2019 (35)^3^	18 June 2019 (5.5)^3^	7 Nov 2019	2 Sep 2019

^1^Based on data collected in Matsumoto et al. (1,4), ^2^All households reported 100% mortality in their herds, ^3^Median (interquartile range)

**Table 2:** Population and mortality data on the ASF-affected villages modelled

Province, District	Village name	Modelled pig population (mortality %)^1^	WAHIS reported pig population (mortality %)^2^
Savannakhet, Thapangtong	Densateung	225 (100%)	655 (8%)
Savannakhet, Thapangtong	Phouphanang-Khampia	182 (100%)	751 (2%)
Oudomxay, Nga	Doneant	43 (100%)	101(55%)
Oudomxay, Nga	Huaylerm	60 (100%)	169 (99%)

^1^Number of pigs to be simulated in the model, based on population data collected in Matsumoto et al. (1,4); ^2^Pig population and mortality % reported to WOAH and available on WAHIS

In Savannakhet, up to twenty-five villagers per village were selected randomly from a list of pig farmers provided by the village chief and surveys were completed between September and October 2019, a few months after the initial ASF outbreak. In Savannakhet, the pig population had not recovered at the time of the survey and there was no outbreak occurring at that time. All villagers were included as potential ASFV-positive households based on the selection criteria outlined by Matsumoto et al. (Matsumoto et al. 2021), where a list of farmers was drawn up by the local Village Chief prior to the arrival of the investigators, and twenty-five names were randomly selected using the random number generator in Excel. These farmers were then asked to make themselves available for the surveys. In one instance, a farmer from Savannakhet was unable to attend and a replacement could not be found. Investigators in Oudomxay approached all available households that reported losses of pigs in the period from March 2019 to March 2020 for the same survey (n = fourteen in Doneant and n = sixteen in Pangthong) (Matsumoto et al. 2023). The surveys in Oudomxay were delayed until December 2020 due to travel restrictions associated with the SARS-CoV-2 pandemic. The survey questionnaire comprised of 28 questions about the household, pig herd demography and estimated monetary value immediately prior to the ASF outbreaks, pig management techniques and ASF outbreak data such as morbidity/mortality rates, estimated date of clinical signs and mortality. All villagers who could not remember the exact dates worked with local PAFO and DAFO investigators to compile the approximate dates of their pigs’ deaths and clinical period from development of clinical signs to death (Matsumoto et al. 2021, 2023). Table 1 demonstrates the styles of housing used in the study sites, with substantially lower amounts of containment (pens or housing) in the Savannakhet villages in comparison to the Oudomxay villages. The median epidemic day was the midpoint of the epidemic, at which half of all the cases had occurred.

### Disease simulation modelling approach

#### Model structure

The ASF outbreak was modelled using the deSolve package in RStudio (Soetaert et al. 2015; RStudioTeam 2018). A Susceptible-Exposed-Infectious-Removed (SEIR) model was developed to replicate ASF spread between-pigs in each of the villages individually, allowing for variations in pig management or environmental factors to be incorporated into final transmission parameter outputs (Keeling and Rohani 2011, pp. 41–43). The modelled host population was assumed to be a sample or a census of all pigs present during the outbreak based on the outbreak investigation surveys (Matsumoto et al. 2021, 2023) (Table 2). Since there were discrepancies between the Government reported mortalities and those recorded in the outbreak investigation surveys, it was decided that there should be an assumption of 100% mortalities by the end of model simulations. The use of a differential approach meant that the compartments were represented as proportions of the population rather than numbers of cases.

**Fig. 1:**
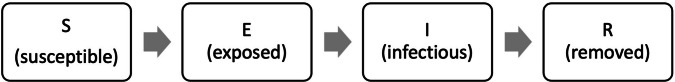
A schematic diagram of the movement of pigs through disease compartments in an SEIR disease model

In a SEIR model, all pigs in a disease affected population will fall into one of the following compartments: Susceptible, i.e. yet to be infected by ASFV; Exposed, i.e. infected with ASFV but not yet showing clinical signs or infectious (often referred to as the latent period); Infectious, i.e. shedding ASFV at high enough levels to infect other Susceptible pigs and that could be showing clinical signs; and Removed, i.e. pigs that have died from ASF and are removed from the population (Fig. 1).

The force of infection (λ) at time *t* indicates the per capita rate at which susceptible individuals become infected and is calculated as:

1 $${\uplambda }_{t} =\beta \frac{{I}_{t}}{{N}_{t}}$$

where *β* is the transmission rate, *I*_*t*_ is the number of infectious pigs, and *N*_*t*_ is the total available population at time *t* (Keeling and Rohani 2011, p. 17). Time was measured in daily time steps for the purposes of this model.

The differential equations for movement between compartments in the SEIR model are shown in Eqs. 1–4. The compartments S, E, I and R represent proportions of the total population (Keeling and Rohani 2011).

2 $$\frac{dS}{dt}= -\beta SI,$$

3 $$\frac{dE}{dt}= \beta SI-\sigma E,$$

4 $$\frac{dI}{dt}= \sigma E- \gamma I,$$

5 $$\frac{dR}{dt}= \gamma I,$$

where 1/σ is the mean duration of the latent period (µ_e_), and 1/γ is the mean duration of the infectious period (µ_i_) (Keeling and Rohani 2011). These parameters (σ and γ) represent the probability of moving from one compartment to the next at each time step. These probability distributions were assumed to follow gamma distributions using the parameters mean and shape. In this study the gamma distributions utilised means µ_e_ and µ_i_ and shapes k_e_ and k_i_ for latent and infectious respectively (Keeling and Rohani 2011; Guinat et al. 2018). In this study, only k_e_ was estimated since existing infectious period data provided information about the k_i_.

The formula for R_0_ in a SEIR model is derived using *β* and the mean infectious period (1/γ) (Eq. 6) (Keeling and Rohani 2011, p. 97).

6 $${R}_{0}=\upbeta \frac{1}{\upgamma }$$

SEIR models assume a closed population with no additional births or deaths in the outbreak period, homogenous (uniform) mixing of all animals in the population, and that the likelihood of coming into contact with another pig is independent of the remaining population size (Keeling and Rohani 2011). It was assumed that the rates of free-ranging in the villages should allow the populations of pigs to be treated as a single group rather than numerous individual household pens (Guinat et al. 2016a; Holt et al. 2019; Matsumoto et al. 2021).

#### Adaptation of field data for modelling

Some farmers did not remember the exact date of their pigs’ deaths but could name a week or a month in which the event occurred. For use in modelling, these mortalities were randomly distributed across the week or month identified by the farmer to avoid creating false peaks in the mortality data using a uniform random probability for the dates in that week or month in Rstudio (RStudioTeam 2018).

The time over which the outbreaks were modelled in days was calculated based on the first and last mortalities, with an additional two latent periods and infectious periods using the upper 95th percentile of the priors. This approach provided ample lead-in time for the model to estimate when the first animal was infected.

### Parameter estimation using approximate Bayesian computation with sequential Monte Carlo

#### ABC-SMC using mortality data

Approximate Bayesian Computation with Sequential Monte Carlo (ABC-SMC) is a stepwise approach to estimating a parameter or a group of parameters. The ABC-SMC model is set up over 15 generations, simulating different variations of parameters (listed in Table 2) by taking them from an informative prior group of estimates with a set level of probability for each value. This group of parameters is referred to as a particle. Each generation of particles are then selected for best replicating the field data by reducing tolerance for the maximum sum of squares between the daily mortalities in the field data and the simulated data, and this is referred to as the threshold sum of squares for that generation (Toni et al. 2009) (Eq. 7).

7 $$sum of squares= \sum_{{t= t}_{1}}^{{t}_{2}}{({mortalities}_{simulated(t)}-{mortalities}_{field(t)})}^{2}$$

Each parameter is drawn from a prior probability distribution in the first generation. In subsequent generations, the parameters are drawn from the previous generation of particles and slightly perturbed (altered) to form the particle for that simulation. The SEIR model is then run using the particle. If the final mortality outputs of the SEIR model were within the threshold for the sum of squares in that generation (named epsilon in Table 3), the particle would be kept for use in the subsequent generation until 10,000 particles were generated. At the end of each generation, the tolerance for the sum of squares was set to the 75th percentile of the sum of squares in the previous generation (McKinley et al. 2009; Toni et al. 2009; Guinat et al. 2018).

**Table 3:** Posterior distributions for key ASF disease transmission parameters in Lao villages using ABC-SMC methods

Village, Province	R_0_ (median, 95% CI)	β (median, 95% CI)	k_E_ (median, 95% CI)	µ_E_ (median, 95% CI)	µ_I_ (median, 95% CI)	ε
Densateung, Savannakhet	6.17 [6.15, 6.18]	1.10 [1.09, 1.10]	16.40 [16.31, 16.47]	6.19 [6.17, 6.20]	5.50 [5.48, 5.51]	0.42
Phouphanang-Khampia, Savannakhet	7.80 [7.78, 7.81]	1.66 [1.65, 1.66]	16.26 [16.18, 16.33]	4.72 [4.71, 4.73]	4.73 [4.71, 4.73]	0.13
Doneant, Oudomxay	3.08 [3.06, 3.09]	1.18 [1.17, 1.19]	16.35 [16.26, 16.42]	5.38 [5.36, 5.39]	2.63 [2.61, 2.64]	3.61
Huaylerm, Oudomxay	5.98 [5.92, 6.04]	1.28 [1.27, 1.29]	16.39 [16.31, 16.46]	5.41 [5.38, 5.42]	5.01 [4.94, 5.06]	0.91

ε – mean sum of squares in final round

After the ABC-SMC model, each parameter was inspected for final distribution and stabilisation of the estimates/marginal distributions for the ABC-SMC model. The final estimates were then used to create an SEIR model to assess the goodness of fit. The model was cut off at fifteen generations because all models stabilized at this round.

#### Transmission parameters used in ABC-SMC approach

Informative prior probability distributions for the outbreak were adapted from Guinat et al. (Guinat et al. 2016a; b, 2018) and the field data in Matsumoto et al. (Matsumoto et al. 2021, 2023). The priors used for each parameter can be found in Table 4. In the original Guinat et al. paper (Guinat et al. 2018), the informative priors were compared with wide uniform priors to determine their impact on the final model, and it was found that the wide uniform priors provided closer fits to the outcome data but took substantially longer to run, with similar output values. For this reason, the wide uniform priors were not included in the results of this paper.

**Table 4:** Informative and non-informative prior data utilised in modelling of transmission parameters

Parameter	Symbol	Informative prior (mean, shape)	Source(s)
Between-pig transmission rate (per day)	*β*	2.00, 2.00	(Guinat et al. 2016a, 2018; Hu et al. 2017; Korennoy et al. 2017)
Shape parameter—latent period	*k*_*E*_	19.39, 5.00	
Mean latent period (days)	*µ*_*E*_	6.08, 19.20	
Mean infectious period (Savannakhet, days)	*µ*_*I*_	5.40, 15.17	(Olesen et al. 2017; Guinat et al. 2018; Stevenson et al. 2019; Matsumoto et al. 2021)
Mean infectious period (Oudomxay, days)		5.74, 1.56	(Olesen et al. 2017; Guinat et al. 2018; Stevenson et al. 2019; Matsumoto 2022)

Infectious period data were collated using the time each villager observed clinical signs. Then an additional twenty-four hours (one day) was added to account for a subclinical infectious period (Olesen et al. 2017). The Savannakhet field data was used to model the villages in Savannakhet, and the Oudomxay field data was used to model the villages in Oudomxay. Both sets of field data were adapted into gamma distributions using the gamma.buster() function of the EpiR package in R (RStudioTeam 2018; Stevenson et al. 2019; R Core Team 2022).

## Results

### Parameter estimates

Due to the parameters being estimated using Bayesian methods, all transmission parameter results are reported with a 95% credible interval instead of a 95% confidence interval. In addition, all posterior distributions were skewed, and the median results are reported here.

After performing ABC-SMC on the transmission parameters used in a SEIR disease model for each village of pigs separately, the posterior outputs for the transmission parameters between-pigs in each separate village are shown in Table 3. Doneant (Oudomxay province) had the lowest R_0_ (3.08) whilst Phouphanang-Khampia (Savannakhet province) had the highest R_0_ (7.80). The transmission rates (β) between-pigs in each of the villages ranged from a median of 1.10 to 1.66. The shape parameter for the latent period (k_E_) did not vary greatly (16.26 to 16.40). The predicted mean latent period (µ_E_) ranged from a median of 4.72 to 6.19 days between-pigs in each of the villages. The predicted mean for the infectious period (µ_I_) ranged from 2.63 to 5.50 days in the posterior distributions (Table 3).

### SEIR model outputs

**Fig. 2:**
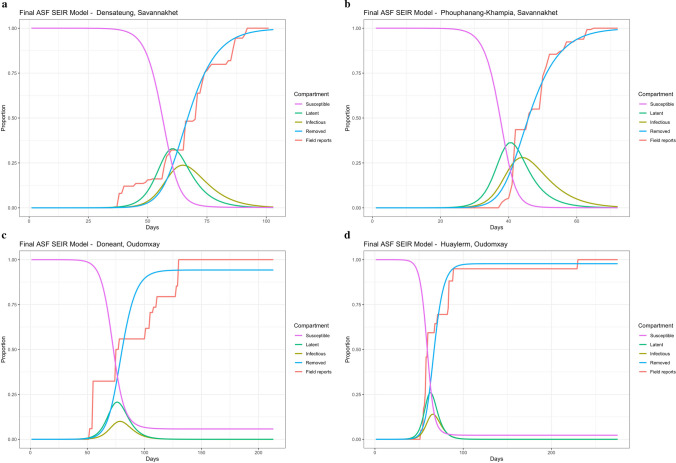
SEIR curve for simulated ASF data overlaid with field ASF daily mortality data in 2019. **a** Densateung, Savannakhet; **b** Phouphanang-Khampia, Savannakhet; **c** Doneant, Oudomxay and **d** Huaylerm, Oudomxay

The final median outputs were utilised to build differential SEIR models which are graphed in Fig. 2. The final cumulative mortality proportion in the field data was within 95% of the posterior distribution in all outputs, as can be seen by the close relationship between the cumulative mortality in the field data and the R (dead) line in the SEIR plots.

## Discussion

By estimating R_0_ in these Lao villages, unique information is presented about how pigs interact (and therefore spread disease) in the smallholder context. Very little is known about these core transmission parameters in smallholder pigs, with most models referring to the same group of experimental and field studies performed in European contexts (de Carvalho Ferreira et al. 2013; Pietschmann et al. 2015; Guinat et al. 2016a; Iglesias et al. 2016; Korennoy et al. 2017). Experimental studies often have strong internal validity, but their external validity falters particularly when applying these assumptions to Southeast Asian and developing countries. By understanding how ASFV spreads amongst pigs in the Lao context, epidemiologists can formulate recommendations for interventions and monitoring that are regionally appropriate.

Upon initial development of the model, it was unknown whether pigs in a smallholder village should be treated as a group of pigs living together and mixing homogeneously in one pen for modelling purposes with direct transmission (i.e., nose-to-nose contact) or as separate pens for each household and primarily indirect transmission of disease. Indirect transmission occurs when the disease is not *directly* spread from one pig to the next (Dohoo et al. 2009). An example of indirect transmission would be small amounts of ASFV being picked up by pigs in contact with blood on contaminated farm equipment or swill feed made from infectious pigs. In this study, it was decided that the rates of free-ranging in the villages justified treating each village as a population of homogeneously mixing pigs, similar to within-pen estimates for other papers. For this reason, further research on the behaviour of free ranging pigs in the Southeast Asian smallholder system and contacts between households is warranted.

Lao smallholder village R_0_ estimates reported here are at the lower end of published estimates for the within-pen R_0_ of commercial breed pigs. Estimates for the ASF within-pen R_0_ vary, but experimentally values from 2.80 to 5.30 have been demonstrated (Guinat et al. 2016a). In field data, estimates for R_0_ become much higher at 4.40 to 17.30 in the Russian Federation and 4.83 to 11.90 in China (Guinat et al. 2018; Li et al. 2021). In the aforementioned studies, it was likely that the direct transmission was being exacerbated by biosecurity and management factors such as workers moving through pens (Guinat et al. 2018). The predicted R_0_ in this work did not follow the trend wide variation between sites as reported by Guinat et al. (Guinat et al. 2018). The R_0_ estimates presented here (3.08 to 7.80) are consistent with the findings of other studies, with the lack of high values possibly suggesting that animals may not interact at the same density as those recorded in the field (Guinat et al. 2016a, 2018; Iglesias et al. 2016; Li et al. 2021). It appears that whilst smallholder free-range pigs do easily contact one another, either management factors or behavioural factors provide a dampening effect on the rate of transmission, and therefore R_0_.

Based upon these results, it is likely that the assumption of pigs mixing homogeneously in the smallholder village needs to be treated with caution as many compartmental disease models make a baseline assumption of homogenous mixing between individuals (Keeling and Rohani 2011; Guinat et al. 2018). Many farmers in these same study groups reported keeping their pigs in pens at night but allowing free-range during the day or utilising containment systems, and spatial clusters were observed in the outbreaks (Matsumoto et al. 2021). In a study of wild pigs in North America, social group membership and distance between groups were identified as crucial factors for effective disease contacts (Yang et al. 2021). Wild pigs maintain multigenerational family social structures of sows and piglets, which may temporarily mix around central resources such as watering points (Cowled and Garner 2008; Podgórski et al. 2014; Yang et al. 2021). Most farmers in the populations studied have reported keeping a sow and her piglets during retrospective investigations of the same outbreaks, and it could be assumed that these household family groups behave similarly to wild pig groups (Matsumoto et al. 2021, 2023). This likely heterogeneous mixing must be considered for future disease spread modelling. Detailed investigation of the social contact structures of smallholder owned domestic pigs would be of huge value in estimating this heterogeneity, as previous work shows evidence of contact between different households’ pigs and wild boar. However, these contacts were not quantified in-depth (Matsumoto et al. 2021, 2023). Future work may also explore the biosecurity of smallholder management strategies such as penning at night-time and their impacts on ASF transmission through prospective study designs or survival analysis techniques.

Compared to experimental and field data on commercial pigs, the smallholder village pigs appeared to have similar latent and infectious periods, except for Doneant (Oudomxay province) where the infectious period was shorter (2.63 days). The retrospective nature of the data collection relied on farmer recall which may have been biased and created inaccuracies in the estimates, so it is therefore reassuring that the raw field data and the estimated parameters mirror the values reported in the literature. Typically, Georgia 2007 and China 2018 studies report latent periods in domestic pigs ranging from four to eleven days and infectious periods of three to fourteen days (Guinat et al. 2016a; Li et al. 2021) depending on the nature of the disease contacts. Many experimental studies only observe fit, non-neonatal individuals, so that the ability of these study individuals to fight an infection may be higher than the animals observed in the current study (Pietschmann et al. 2015; Guinat et al. 2016a; Li et al. 2021). The population structure dominated by piglets presents a plausible source for the variation in the infectious period between the estimates for Doneant and the other sites. Herds in these groups were inherently biased to piglets due to the population structures of these herds – for example Oudomxay province farmers owned an average of two sows and six piglets (Matsumoto et al. 2023). Piglets rely upon their mother for nourishment and hydration, and the impact of the sow becoming viraemic and producing less milk, will be starvation and dehydration, hastening the onset of death. This might be reflected in the shorter estimate for the infectious period. In the outbreak investigations, a small number of households reported mortalities in more detail, with their piglets dying within a faster time frame than their sows and boars: two to four days for piglets and six to seven days for sows (Matsumoto et al. 2021). Ultimately, the results for the Doneant site should be interpreted with caution due to the poorer model fit when compared with the other sites – as demonstrated by the higher value for epsilon. Future work with larger population sizes would allow separation of the modelling into age groups, family groups and genders to estimate their impacts on how disease occurs in these different groups.

Across all modelled villages, the similarities in transmission parameters suggest that despite diverse household management techniques (Matsumoto et al. 2021, 2023), pig contact structures are relatively consistent between Lao smallholder villages. This consistency is highly valuable for future modelling and decision-making purposes as recommendations can be made at the whole-village level. Future studies should assess disease transmission parameters across various ethnic and geographic scenarios to account for the possibility of village-level selection bias. A wide sampling frame of different villages would highlight special ethnographic or geospatial considerations when modelling at higher population levels such as whole provinces or districts.

Understanding the genotype of a newly invading pathogen allows epidemiologists to draw inferences about the origin and behaviour of an ASF outbreak. The similarities in the latent periods between modelled villages (4.72 to 6.19 days) indicate that these outbreaks might have been caused by the same genotype of the ASFV. Based on the geographic disparity between these locations (934 km between Oudomxay and Savannakhet provinces), human-assisted spread can be assumed in the national outbreak from village to village. In the case of Laos, only Oudomxay province shares a border with China, whilst Savannakhet lies between Thailand and Vietnam. The Vietnamese outbreak in 2019 demonstrated shared identical genotypic characteristics with the Georgia 2007 and China 2018 isolates (Le et al. 2019). If there had been different disease parameters, the assumption could be made that different strains were spreading in the villages of Oudomxay compared to the villages of Savannakhet.

At the village level, selecting surveyed households at random or as a census reduces or negates, respectively, the possibility of selection bias in the host populations. There was some uncertainty about the true mortalities and numbers of households affected in the Government reporting, and as such the data used in the models were collected from whole villages chosen purposively for the study due to location (northern and southern for Oudomxay and Savannakhet, respectively). This must be accounted for when making assumptions about the outputs of this model and their generalisation to the population at large, but data on the pigs and households themselves are likely representative of each village (Matsumoto et al. 2021). Sensitivity testing of the host population structure – in particular the mortality rates – would provide better insight into the relative impact of this uncertainty of host structures in the final parameter estimated. Repeating this work in numerous contexts would also provide a less village-biased group of parameter estimates.

The disease spread modelling approach was simplistic due to the two-part nature of this study, where the SEIR model was run 10,000 times in each generation of the ABC-SMC process. The model was designed without background births, deaths, additions, or removals. The model also assumed that dead pigs were appropriately disposed of rather than left available for other pigs to cannibalise and become exposed to the ASF virus after their removal from the population. This model choice may have oversimplified transmission probabilities. Future disease models in the Lao context should account for factors such as a variable subclinical infectious period and heterogenous contact structures between pigs. For example, a sow is much more likely to contact her piglets than another sow, and this should be reflected in future models (Vynnycky and White 2010; Keeling and Rohani 2011; Brauer and Castillo-Chávez 2013).

The outcomes and challenges of this work provides useful baseline information for local epidemiologists and policymakers for parameterising models that can be used in future decision-making about effective strategies for preventing an outbreak of ASF or similarly high-impact disease in smallholder village settings. It demonstrates the need for additional support of local veterinary authorities in not only managing, but accurate surveillance and reporting of outbreaks. To fully define effective management strategies, the data and techniques presented in this paper could be utilised to estimate transmission parameters between villages at the national level. It is possible that once the disease is in a village, the rate of transmission to other villages is much faster, and so resources should be focused upon the prevention of ASF spread between villages. Conversely, the slow rate of transmission within villages may suggest that the most effective strategy for ASF prevention is to prevent spread between households in an infected village. Education programmes that support smallholder farmers to strengthen on-farm biosecurity is recommended. Future studies could look at similar parameters between whole villages. By adopting high-quality field data to locally relevant disease parameter estimates, these recommendations will aid in the protection of smallholder food and income security from disease outbreaks in Lao and Southeast Asian contexts.

## Data Availability

Data can be made available upon reasonable request.
